# Self-supervised and few-shot learning for robust bioaerosol monitoring

**DOI:** 10.1007/s10453-025-09850-4

**Published:** 2025-04-09

**Authors:** Adrian Willi, Pascal Baumann, Sophie Erb, Fabian Gröger, Yanick Zeder, Simone Lionetti

**Affiliations:** 1 Department of Computer Science and Information Technology, Lucerne University of Applied Sciences and Arts, Suurstoffi 4, 6343 Rotkreuz, ZG Switzerland; 2 Surface Measurements, Federal Office of Meteorology and Climatology MeteoSwiss, Chemin de l’Aérologie 1, 1530 Payerne, VD Switzerland; 3 Environmental Remote Sensing Laboratory, École Polytechnique Fédérale de Lausanne, Station 2, 1015 Lausanne, VD Switzerland; 4 Department of Biomedical Engineering, University of Basel, Hegenheimermattweg 167b, 4123 Allschwil, BS Switzerland; 5 Swisens AG, Meierhofstrasse 5a, 6032 Emmen, LU Switzerland

**Keywords:** Pollen, Holographic imaging, Self-supervised learning, Few-shot learning

## Abstract

Real-time bioaerosol monitoring is improving the quality of life for people affected by allergies, but it often relies on deep learning models which pose challenges for widespread adoption. These models are typically trained in a supervised fashion and require considerable effort to produce large amounts of annotated data, an effort that must be repeated for new particles, geographical regions, or measurement systems. In this work, we show that self-supervised learning and few-shot learning can be combined to classify holographic images of pollen grains using a large collection of unlabelled data and only a few identified particles per type. We first demonstrate that self-supervision on pictures of unidentified particles from ambient air measurements enhances identification even when labelled data are abundant. Most importantly, it greatly improves few-shot classification when only a handful of labelled images are available. Our findings suggest that real-time bioaerosol monitoring workflows can be substantially optimized, and the effort required to adapt models for different situations considerably reduced.

**Supplementary Information:**

The online version contains supplementary material available at 10.1007/s10453-025-09850-4.

## Introduction

Real-time aerosol monitoring provides key information for public health (Buters et al., 2022). Pollen is among the most common causes of respiratory allergies in European countries, making pollen monitoring key to diagnosing, managing, and treating symptoms. Although Hirst-type impactors (Hirst, 1952) have been providing reliable airborne pollen data for decades, they have limitations such as low sampling rates (10 l/min), measurement uncertainties, processing delays, and labour-intensive operations.

Recent advances in laser and artificial intelligence technologies address some of these limitations and have led to the development of new automatic bioaerosol monitoring instruments (Huffman et al., 2020). The SwisensPoleno is one such new system based on airflow cytometry (Sauvageat et al., 2020). It measures particles through holographic imaging, laser-induced fluorescence, light scattering and polarization. These measurements are then used to classify airborne particles using deep learning models. To date, this has been carried out in a setting which requires substantial amounts of labelled data. The annotation of this data needs to be performed by experts and is labour-intensive, and thus often constitutes a bottleneck. Another limitation of this approach is that models developed for a certain geographical region can perform poorly in other regions where atmosphere composition is different. To classify new types of bioaerosol not included in the training data, it is typically necessary to re-train models. Current research, including European efforts like the EUMETNET AutoPollen Programme and the SYLVA Horizon Europe project, are developing models, networks, and datasets at the European level to address this issue. Nevertheless, a complementary approach is to reduce the need for labelled data covering geographical regions, measurement systems, atmospheric and operational conditions.

In this paper, we focus on the identification of ambient particles based on holographic images acquired by the SwisensPoleno. Starting with a general-purpose deep learning model trained on ImageNet with supervision, we refine it on 20 million unlabelled images of airborne particles with a self-supervised learning method called SimCLR. We then consider pollen as a case study because of its suitability for holographic imaging, the availability of labelled data, and its relevance to public health. We investigate if model refinement with self-supervision leads to enhanced robustness to variations in data acquisition settings. More precisely, we study generalization in the setting where one device is used for training and a different one for evaluation, mimicking the addition of a new measurement system in a monitoring network without additional training. This is a limited scenario compared to all the effects that could compound in production, but still gives a first indication of non-trivial distribution shift. Finally, we explore the combination of self-supervised training with few-shot learning for particle identification when only a small number of labelled particles are available.

## Methods

This study leverages deep learning to process automatically captured holographic images of ambient particles. Generalized and robust representations of unlabelled images are constructed using self-supervised learning (SSL). Subsequently, these representations are combined with few-shot learning (FSL) to improve classification performance using only a minimal set of labelled samples.

### Data

The SwisensPoleno instrument (Sauvageat et al., 2020) takes in-flight holographic images of particles as they flow through the measurement system. Passing particles with sizes between 5 and 200 µm trigger two cameras, which are perpendicular to each other and to the particle flow. Their raw holographic images are reconstructed in focus, centred, and cropped to 200$\times$200 pixels grey-scale pictures where one pixel corresponds to 0.595 µm.

In this study, we use both unlabelled and labelled data. The unlabelled data consist of about 20 million holographic images of ambient airborne particles measured by a SwisensPoleno located on the rooftop of the MeteoSwiss building in Payerne, Switzerland, throughout one year. The labelled data were obtained from specific measurements during which pollen collected directly from known plants was aerosolized. These data were then manually filtered and cleaned as described in previous work (Erb et al., 2023). Eleven different plant taxa were considered—*Alnus glutinosa (L.) Gaertn., Carpinus betulus L., Cupressus sempervirens L., Cynosurus cristatus L., Fagus sylvatica L., Fraxinus excelsior L., Picea abies (L.) H.Karst., Populus sp., Quercus robur L., Taxus baccata L.* and *Ulmus glabra Huds*. The labelled data were obtained from two SwisensPoleno systems denoted P4 and P5, and consist of 400 to 6’600 pollen grains per taxon and instrument. Although the large unlabelled collection comes from P5, there is no overlap between the labelled and unlabelled data.

### Machine learning

**Fig. 1:**
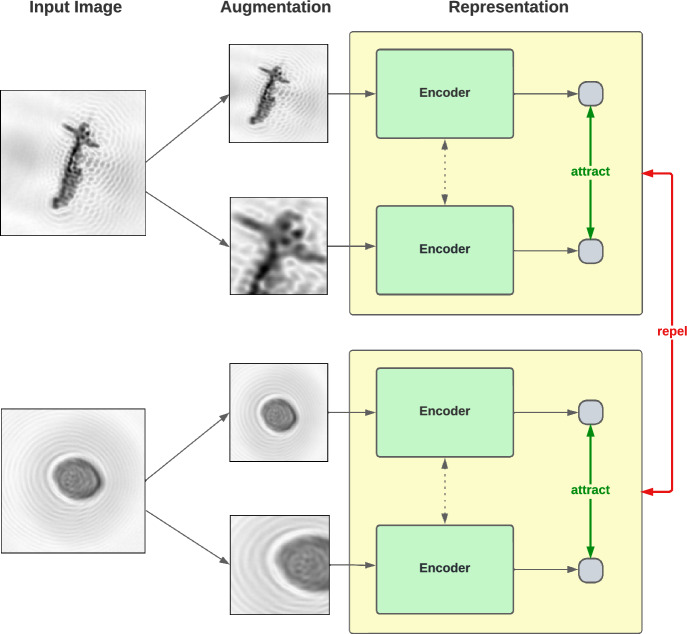
Simplified illustration of the SimCLR framework using holographic images of airborne particles from SwisensPoleno. Every input image in a batch is augmented, resulting in different views of the same particle. These views are then all passed through the same deep neural network encoder to obtain vector representations. Finally, representations from the same input image are attracted, while the ones from different input images are repelled. This produces semantic representations without the need for labelled data

SSL offers a way to build models without labelled data. In particular, SimCLR (Chen et al., 2020), the method used in this work, belongs to the family of contrastive SSL approaches. These methods learn vector representations of images by generating multiple augmented versions for each original image through augmentations like rotation and clipping, and using a deep neural network encoder to map the augmented images into vectors. The encoder is trained by pulling together these vectors if they come from the same original image and repelling them otherwise, as illustrated in Fig. 1. We apply SimCLR to the unlabelled SwisensPoleno holographic images and initialize it with a general-purpose image encoder pre-trained on ImageNet in a supervised way, as this performs better compared to random initialization (Azizi et al., 2023).

To classify new data with a limited number of labelled images, we combine SSL with FSL. First, we test a linear classifier, where we combine image representations with one-vs-all logistic regression. Second, we learn a prototype in representation space for each taxon by minimizing cross-entropy after assigning samples to prototypes with cosine similarity. The latter approach was proven to perform consistently better than the linear classifier and on par with more sophisticated techniques across many FSL scenarios (Chen et al., 2019).

We compare the baseline model trained on ImageNet to our model that has been specialized for holographic aerosol images with SimCLR, always using the same EfficientNet-B0 architecture (Tan & Le, 2019). We measure performance using balanced accuracy which gives the same importance to all taxa, as our dataset does not reflect atmospheric composition.

## Results

We first consider the case where the model is trained on all available labelled data. We use half of the data from one instrument for training and evaluate previously learned representations with one-vs-all logistic regression on the rest of the data. This evaluation setup is still not a full proof of principle using data from different locations, but a step in this direction. Results are presented in Table 1. When evaluating the model trained on data from the same instrument P5, we observe that specializing the supervised ImageNet features on SwisensPoleno data with SimCLR marginally improves the balanced accuracy (+0.5%) compared to ImageNet alone. However, the gain is larger (+3.5%) when evaluation is carried out on data from the other instrument P4. In these conditions, the ImageNet performance drops to 75.5%, while that of ImageNet+SimCLR only drops to 79%.

**Table 1:** Balanced accuracy for the classification of 11 pollen taxa from the features of the ImageNet+SimCLR model and ImageNet, using a linear classifier (one-vs-all logistic regression) trained on a labelled P5 training dataset and evaluated on the labelled P5 and P4 test datasets

	Balanced accuracy score	
Method	P5 test dataset	P4 test dataset
ImageNet	$89.9 \pm 0.4$ %	$75.5 \pm 0.4$ %
ImageNet+SimCLR	$90.4 \pm 0.4$ %	$79.0 \pm 0.4$ %

**Fig. 2:**
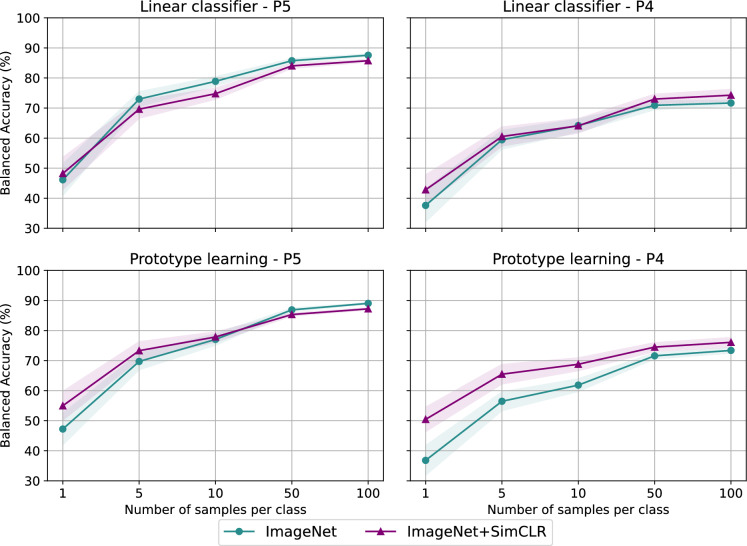
Balanced accuracy for pollen classification as a function of the number of available labelled images per taxon, evaluated on data from the same SwisensPoleno (P5) used for SSL (left), and on data from another SwisensPoleno (P4) (right). Results are shown for the linear classifier (top) and prototype learning (bottom) on top of features from ImageNet (teal) and ImageNet+SimCLR (purple). Coloured bands represent the uncertainty due to the random choice of the labelled images

Next, we address situations where training data are scarce. Figure 2 shows the performance of different methods as a function of the number of labelled images available per taxon. When evaluation is performed on test data from the same measurement system as the training data, i.e. from P5 (left column), ImageNet+SimCLR outperforms ImageNet with only one image per taxon. As the amount of labelled data increases, ImageNet gains an edge over ImageNet+SimCLR, and differences among models become smaller. If less than 10 images per taxon are available, the best results are obtained by combining ImageNet+SimCLR with prototype learning, while the ImageNet model does not profit from this FSL technique. When the evaluation is performed on test data from a different measurement system than the training data, i.e. P4 (right column), ImageNet+SimCLR outperforms ImageNet throughout. Moreover, the synergy of ImageNet+SimCLR and prototype learning yields significantly better results compared to the other three alternatives. When a single example is available, this combination achieves 50% balanced accuracy, while all other methods are around 40%. With only five or ten labelled examples, one can already expect a balanced accuracy around 65% and 70%, respectively.

In general, we observe significant performance drops when evaluation is performed in a different setting compared to training, even if holographic images exhibit very little variations among measurement systems. These drops are markedly reduced in the case of ImageNet+SimCLR with prototype learning, highlighting the robustness of distance-based classification with self-supervised features. By contrast, purely supervised models are more prone to take shortcuts for classification, which might explain why they generalize poorly when conditions change (Robinson et al., 2021). Therefore, we expect that the combination of SSL with FSL, which reduces the performance mismatch among different measurement systems, will result in improved or at least equivalent performance in an operational setup.

## Discussion and conclusion

ImageNet+SimCLR offers more robust performance on different instruments and sample conditions compared to training, which is already an advantage for pollen monitoring networks. The combination of SSL with FSL can produce flexible identification models, and therefore has an even greater appeal for networks covering regions with broad aerobiological diversity. SSL requires additional training compared to off-the-shelf models, but this only needs to be done once and taxa can be specified a posteriori. Indeed, our simplified setup suggests that SSL does not need to be repeated if the large unlabelled data collection provides sufficient coverage of particle types. To transfer models across environments, FSL should be performed with a small amount of local data for all relevant particles, multiple measurement systems, and different environmental conditions. Hence, the significant potential benefit for the development of new pollen identification models is that the effort of labelling pollen data may be reduced to a handful of particles, if performance remains within acceptable limits. This opens the door to faster development of models across climatic regions and may be even more helpful for particle types which are very challenging to label, such as fungal spores. It should be noted that a validation of the improved classification performance with the manual standard EN16868 is still missing and requires a dedicated measurement campaign, since the setup to collect labelled pollen grains does not lend itself to this comparison.

In this study, we showed that self-supervision can effectively leverage large volumes of unlabelled data to improve the robustness of deep learning models for pollen grain identification against changes in data collection conditions. The approach makes data preparation more feasible and produces flexible models, since other bioaerosol types can be classified with FSL as long as the SSL training dataset is sufficiently diverse. Results could be improved further by training the model on data which covers different collection conditions, including several instruments of the same type and different geographical regions. Similarly, an improvement is also expected when incorporating other measurements from the same system, such as fluorescence and polarization besides holography. Additional information is indeed expected to outweigh measurement variability in between systems. Future work should include testing other SSL methods, especially non-contrastive ones (Grill et al., 2020), and merging current research efforts to develop a foundation model handling a large set of bioaerosol.

## Supplementary Information

Below is the link to the electronic supplementary material.

Supplementary file 1 (pdf 155 KB)

## Data Availability

The data presented in this paper were kindly provided by the Federal Office of Meteorology and Climatology MeteoSwiss, and cannot be shared publicly.

## Declarations

### Code availability

The code used in this work is experimental and tailored to the SwisensPoleno. Considering that it cannot be used without the specific data to reproduce results nor for applications with other devices, there is little benefit making it open-source.
