# Supplementary material for: Self-supervised and few-shot learning for robust bioaerosol monitoring
Source: Aerobiologia (Bologna). 2025 Apr 9;41(2):263–8. doi: 10.1007/s10453-025-09850-4 (PMC12176922; doi:10.1007/s10453-025-09850-4)
Supplement: Supplementary file 1 — (pdf 155 KB) [file 10453_2025_9850_MOESM1_ESM.pdf]

Self-Supervised and Few-Shot Learning for Robust Bioaerosol Monitoring

Adrian Willi, Pascal Baumann, Sophie Erb, Fabian Gröger,
Yanick Zeder, Simone Lionetti

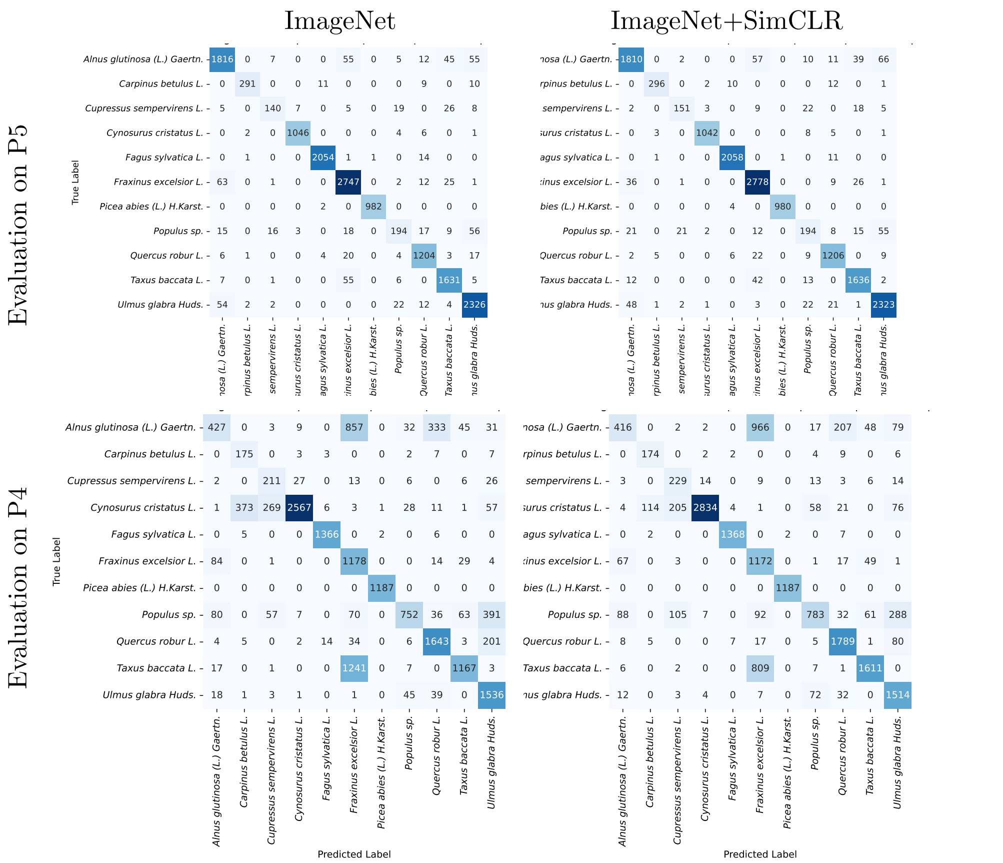

Confusion matrices for the classification of eleven pollen taxa using ImageNet (left column) and ImageNet+SimCLR (right column) features for evaluation sets from the P5 (top row) and P4 (bottom row) measurement systems, as in main paper Table 1.
